# Hypoxia-powered MRI guides supramaximal biosurgical sampling of glioblastoma infiltration: the GLIOXY initiative

**DOI:** 10.1007/s11060-026-05864-1

**Published:** 2026-11-06

**Authors:** Tristan Schmidlechner, Vittorio Stumpo, Elisabeth Jehli, Leonie Zerweck, Meltem Gönel, Jacopo Bellomo, Flavia Müller, Natalia Cantavella-Franch, Martina Sebök, Andrea Bink, Nicolin Hainc, Zsolt Kulcsar, Anna Maria Reuss, Tibor Hortobagyi, Regina R. Reimann, Carlo Serra, Michael Weller, Luca Regli, Christiaan Hendrik Bas van Niftrik, Jorn Fierstra

**Affiliations:** 1 Department of Neurosurgery, Clinical Neuroscience Center, University Hospital, University of Zurich, Frauenklinikstrasse 10, Zurich, CH-8091 Switzerland; 2 Department of Neurology, Clinical Neuroscience Center, University Hospital, University of Zurich, Zurich, Switzerland; 3 Department of Neuroradiology, Clinical Neuroscience Center, University Hospital, University of Zurich, Zurich, Switzerland; 4 Department of Diagnostic and Interventional Neuroradiology, University Hospital Tuebingen, Tuebingen, Germany; 5 Institute of Neuropathology, Clinical Neuroscience Center, University Hospital, University of Zurich, Zurich, Switzerland

**Keywords:** Glioblastoma, BOLD MRI, Imaging biomarker, Targeted biopsy, Supramaximal, Non-enhancing tumor

## Abstract

**Purpose:**

To assess the feasibility of using hypoxia-powered magnetic resonance imaging (MRI) to guide prospective sampling of high-response habitats beyond contrast enhancement in glioblastoma.

**Methods:**

Nine treatment-naive patients with histologically confirmed isocitrate dehydrogenase (IDH)-wildtype glioblastoma underwent blood oxygenation level-dependent (BOLD) MRI during a controlled transient hypoxic stimulus on the evening before surgery. Anatomical segmentation and voxel-wise temporal decomposition generated normalized hypoxia response maps. Otsu thresholding defined a high-response habitat outside contrast enhancement. Targets were confirmed with the surgeon according to anatomical accessibility and eloquence, transferred to neuronavigation, and sampled before major debulking. We divided the specimen for formalin-fixed, paraffin-embedded neuropathology and frozen-tissue storage.

**Results:**

Neuronavigation transfer succeeded in all nine cases. Eight targets were accessible and sampled. Seven sampled targets contained tumor. One yielded reactive cortex and white matter without definite tumor. No target-sampling complications were observed among the eight sampled procedures.

**Conclusion:**

Hypoxia-powered MRI could be integrated into prospective neuronavigation-guided sampling beyond contrast enhancement. Tumor was identified in seven of eight sampled targets. By translating a tracer-free physiological response map into coordinate-matched tissue, the GLIOXY workflow enables prospective, targeted tissue sampling of non-enhancing glioblastoma regions.

**Supplementary Information:**

The online version contains supplementary material available at https://doi.org/10.1007/s11060-026-05864-1.

## Introduction

Glioblastoma remains associated with frequent recurrence and poor survival [1, 2]. The appropriate surgical extent beyond contrast enhancement remains unresolved [3, 4]. Contrast enhancement reflects blood-brain barrier disruption and remains the principal imaging target for resection and radiotherapy [5, 6]. However, glioblastoma infiltrates into non-enhancing fluid-attenuated inversion recovery (FLAIR)-abnormal tissue, where residual disease may contribute to progression [7–9].

Retrospective studies suggest that resection beyond contrast enhancement may improve outcome in selected patients, but definitions based on the volume of FLAIR abnormality do not account for spatially heterogeneous invasion [3, 4, 6–10]. It remains uncertain which parts of this compartment warrant targeting, preservation, or local treatment. Response Assessment in Neuro-Oncology groups have therefore called for standardized imaging-informed sampling of non-contrast-enhancing presumed tumor [11]. Recent prospective work used artificial intelligence-derived maps to target regions beyond enhancement. Histopathology, transcriptomics, and survival analysis supported the biological and clinical relevance of spatial infiltration mapping [12].

Hypoxia-powered magnetic resonance imaging (MRI) uses blood oxygenation level-dependent (BOLD) imaging to measure the regional response to a controlled transient hypoxic stimulus [13–15]. The stimulus increases deoxyhemoglobin, which changes susceptibility contrast and lowers the BOLD signal. Baseline blood volume and oxygen extraction both influence response magnitude [16, 17]. Abnormal microvascular transit and altered metabolic demand in glioblastoma may reduce effective oxygen extraction and increase the response to arterial desaturation [17]. This provides a rationale for investigating high-response regions beyond enhancement as possible sites of altered tumor-associated physiology. The response is not a direct measure of tissue hypoxia or a specific marker of infiltration [17]. We hypothesized that high-response regions beyond enhancement could identify tissue suitable for targeted biological investigation. Whether such regions are enriched for infiltrating tumor over low-response FLAIR tissue remains unproven. Voxel-wise temporal decomposition separates response arrival, descending and recovery transitions, and plateau response magnitude [18]. A related imaging-only study characterized response magnitude and heterogeneity across MRI-defined glioblastoma habitats but did not evaluate prospective surgical targeting or target-linked tissue [19].

**Fig. 1:**
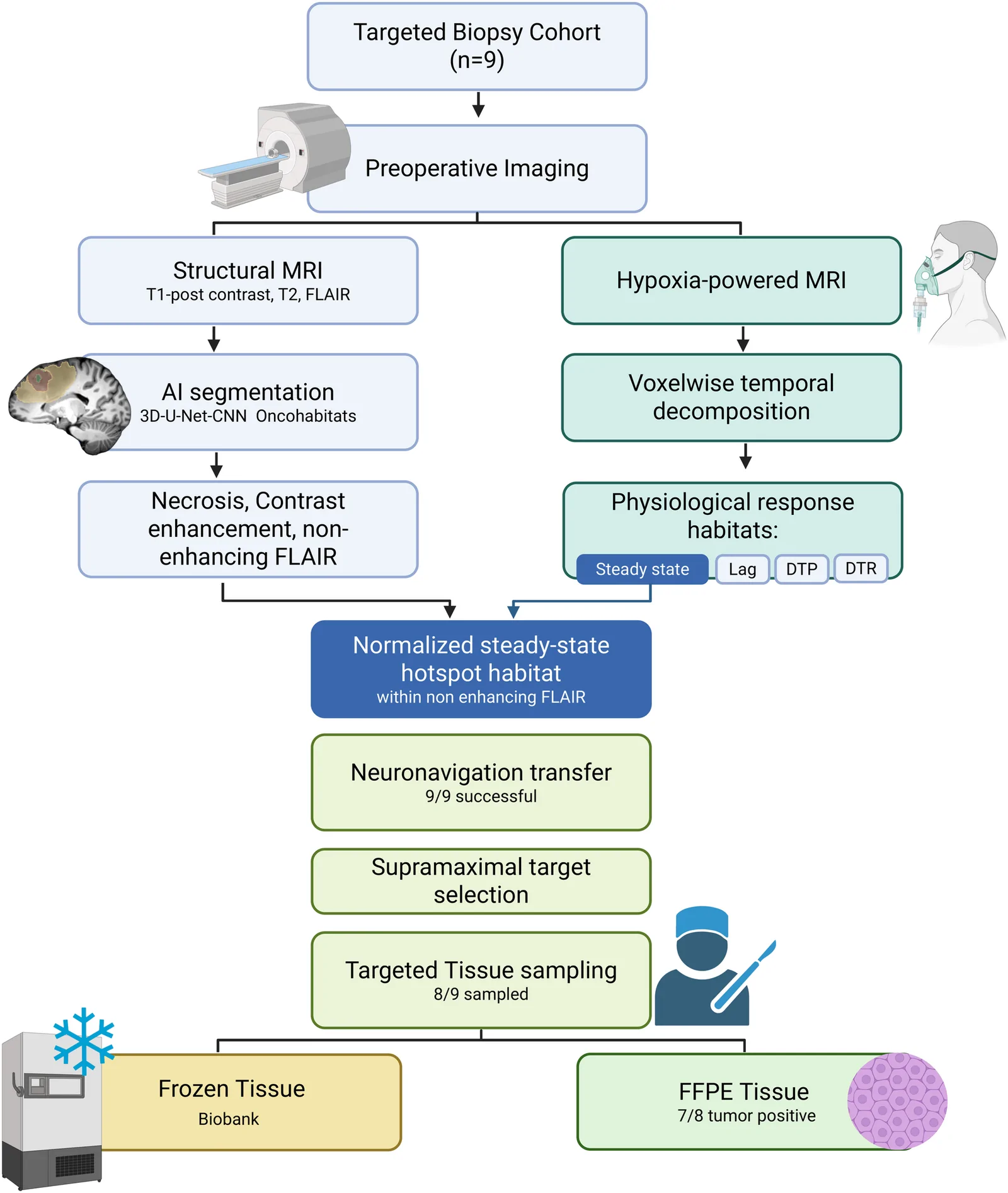
Imaging-to-tissue workflow and habitat definition. Anatomical segmentation and voxel-wise BOLD mapping defined a normalized high-response habitat using Otsu thresholding. Targets were required to lie outside contrast enhancement and were confirmed with the surgeon according to anatomical accessibility and eloquence. The response map was transferred to neuronavigation after registration quality control. Lag, delay to plateau (DTP), and delay to return (DTR) served as complementary temporal descriptors. BOLD, blood oxygenation level-dependent, FFPE, formalin-fixed, paraffin-embedded, FLAIR, fluid-attenuated inversion recovery, MRI, magnetic resonance imaging, AI, artificial intelligence, CNN, convolutional neural network, 3D, three-dimensional. Created with BioRender.com

We investigated whether a stimulus-evoked BOLD map could be used to select a high-response habitat outside contrast enhancement, transfer it to neuronavigation, and sample it during surgery. We also described whether the sampled targets contained tumor. The primary objective was operational feasibility, rather than diagnostic accuracy or validation of the biological mechanism. Figure 1 summarizes the workflow.

## Methods

### Study design and patient population

We conducted this prospective, single-center proof-of-concept study at University Hospital Zurich from November 2025 to July 2026. Treatment-naive adults with imaging suspicious for glioblastoma underwent preoperative hypoxia-powered MRI before planned resection. All included tumors were histologically confirmed isocitrate dehydrogenase (IDH)-wildtype glioblastomas. BOLD MRI was performed on the evening before surgery in every patient. Zerweck et al. [19] previously included one participant from the present cohort in an imaging study of region-level BOLD magnitude and heterogeneity. That study did not report the prospective target selection, neuronavigation, feasibility, or target-specific neuropathology outcomes evaluated here.

The workflow (Fig. 1) sampled physiological MRI habitats beyond contrast enhancement. We defined targeted supramaximal biopsy as neuronavigation-guided sampling from a prospectively selected physiological target outside the contrast-enhancing compartment. The term describes biopsy location and imaging basis and does not imply complete resection of surrounding non-enhancing tissue.

The Cantonal Ethics Committee Zurich approved the study (BASEC 2020–02314 and BASEC 2021 − 00652). All participants provided written informed consent.

### Hypoxia-powered MRI

The controlled stimulus and BOLD acquisition followed published brain-tumor protocols [13, 15]. A computer-controlled system (RespirAct Gen4, Thornhill Medical, Toronto, Canada) delivered two 60-second hypoxic steps targeting an end-tidal oxygen partial pressure of 40 mmHg, separated by 40 s of normoxia, while maintaining isocapnia. We acquired images on a 3-T Skyra VE11 with a 32-channel head coil using a 5-minute T2*-weighted gradient-echo echo-planar imaging (EPI) sequence. The repetition time was 1.8 s and the echo time was 30 milliseconds. The acquisition comprised 50 interleaved 2.5-mm slices and 160 measurements. The field of view was 220 × 220 mm and the flip angle was 80 degrees.

We preprocessed the BOLD series and performed voxel-wise temporal decomposition in Statistical Parametric Mapping version 12 (SPM12) and MATLAB R2024b using the iterative framework described previously [18]. The algorithm calculated voxel-specific 10% and 90% thresholds from lag-adjusted baseline and hypoxic-step signal levels. After identifying an initial 90% crossing, it searched backward within the descending transition for the corresponding 10% crossing and recalculated the thresholds until they stabilized. It applied the same procedure in reverse during recovery. The decomposition yielded corrected lag, delay to plateau (DTP), delay to return (DTR), and a voxel-specific steady-state (plateau) interval.

For each voxel, we averaged the BOLD signal change across this plateau interval, normalized it by the achieved end-tidal oxygen step amplitude, and expressed the result as %BOLD change/mmHg. These values formed the hypoxia response map used for targeting. Lag, DTP, and DTR served as complementary temporal descriptors.

### Structural segmentation

We processed structural MRI with ONCOhabitats, whose morphological segmentation uses a three-dimensional U-Net classifier [20]. The automated masks delineated contrast-enhancing tumor, necrosis, and T2/FLAIR abnormality. We used these masks for anatomical context and required formal target eligibility to lie outside the contrast-enhancing contour.

### Hypoxia response map normalization and preparation

Under the applied sign convention, the hypoxic stimulus produced negative BOLD changes. For surgical planning, we therefore inverted the hypoxia response map and normalized each voxel to the median inverted response in contralateral normal-appearing white matter (WM) derived from SPM12 tissue priors. A relative response above 1 indicated a larger response magnitude than the white matter reference.

We rigidly coregistered the hypoxia response map from echo-planar imaging space to the postcontrast T1-weighted image using normalized mutual information, then resliced it with trilinear interpolation. Registration had to pass visual quality control before transfer to neuronavigation.

We converted the resliced hypoxia response map to a derived magnetic resonance series in Digital Imaging and Communications in Medicine (DICOM) format using postcontrast T1 geometry and transferred this physiological series together with the anatomical segmentations to the Medtronic neuronavigation system.

### Prospective physiological habitat and target selection

Before surgery, a patient-specific Otsu threshold separated higher- from lower-response voxels within non-enhancing FLAIR-abnormal tissue on the normalized hypoxia response map. The high-response class defined the candidate habitat. The corresponding focus was identified on the continuous response map. Location outside contrast enhancement was required.

The threshold-defined target was discussed with the surgeon and confirmed by consensus. Signal prominence was determined by Otsu thresholding. Surgical accessibility was assessed from anatomy, eloquence, and the planned approach. Clinical judgment concerned safe access and the final sampling point within the selected region. Unsafe trajectories were not pursued. Interobserver reproducibility was not formally assessed.

### Neuronavigation and intraoperative tissue sampling

The neurosurgeon sampled the target under neuronavigation guidance before major debulking to limit brain-shift error. The final sampling coordinate was recorded. All reported target measurements refer to this recorded sampling location, rather than the planned target. Specimen identifiers linked the sampled target to neuropathology.

### Tissue processing and storage

We divided each specimen. We fixed one portion in formalin and embedded it in paraffin for routine neuropathology and future immunohistochemistry. We froze and biobanked the second portion for molecular analyses.

### Histopathological assessment

Neuropathological assessment was performed as part of routine clinical care and was not blinded to the BOLD imaging findings. A sampled target was classified as tumor-positive when tumor was explicitly documented in a specimen from that target region. For tissue without definite tumor, the diagnostic wording of the report was retained, including reactive changes.

We recorded tumor content or cellularity, Ki-67, necrosis, vascular proliferation, and immunohistochemistry only when explicitly attributed to the target specimen. Original qualifiers, ranges, and upper bounds were retained. Available main-tumor findings were described as routine clinical comparators, not systematically acquired spatially matched controls. We also recorded qualitative O6-methylguanine-DNA methyltransferase (MGMT) promoter methylation status and the available clinical DNA methylation class for cohort characterization.

### Statistical analysis

Habitat volume was determined retrospectively from the Otsu high-response component containing the recorded sampling coordinate. DTP, DTR, and lag were extracted at that recorded coordinate and converted using repetition time = 1.8 s. Target-to-enhancement distance was the minimum three-dimensional Euclidean distance to the nearest contrast-enhancing voxel center and was not calculated when no enhancing compartment was identified. Retrospectively, we compared each recorded target response with an Otsu threshold calculated from finite, sign-corrected, non-WM-normalized steady-state values within the non-enhancing FLAIR mask. We reported the target-to-threshold ratio and the empirical response percentile within this same mask.

Analyses were descriptive. Workflow outcomes were summarized per patient and sampled target. Continuous measures were summarized as medians, interquartile ranges (IQRs), and ranges. No diagnostic-accuracy analysis or between-patient response-pathology association analysis was performed.

## Results

### Cohort and tumor characteristics

The cohort comprised nine patients with IDH-wildtype glioblastoma (median age, 62 years, range, 52–83), including five men and four women. All underwent resection on the day after BOLD MRI. Five tumors involved the temporal or mesial temporal region. The remaining tumors were frontal, postcentral, thalamic, or insular. Eight were left-sided. Median segmented contrast-enhancing volume was 10.43 cm3 (IQR, 2.65–15.34, range, 0.01–35.15), and median non-enhancing FLAIR-abnormal volume was 30.38 cm3 (IQR, 26.87–62.74, range, 9.31–91.83) (Table 1). The MGMT promoter was methylated in five tumors and unmethylated in four. Recorded DNA methylation classes were receptor tyrosine kinase (RTK) I in three tumors, RTK II in three, and mesenchymal in two. The class was unavailable for one tumor (Table 1).

**Table 1:** Patient and tumor characteristics of the proof-of-concept cohort

Case	Age at surgery, y	Sex	Tumor location	CE volume, cm3	Non-enhancing FLAIR-abnormal volume, cm3	MGMT promoter status	DNA methylation class
Case 1	64	Male	Left temporal	20.74	91.83	Methylated	RTK I
Case 2	60	Male	Left mesial temporal	0.34	62.74	Methylated	RTK I
Case 3	55	Female	Left frontal	10.43	66.71	Methylated	RTK I
Case 4	69	Female	Left postcentral	35.15	27.99	Unmethylated	RTK II
Case 5	62	Female	Left mesial temporal	0.01	26.87	Methylated	Mesenchymal
Case 6	52	Male	Left thalamus	7.55	9.31	Unmethylated	RTK II
Case 7	77	Female	Left insular	15.34	30.38	Unmethylated	Mesenchymal
Case 8	83	Male	Left temporal lobe	12.15	55.24	Methylated	Unavailable
Case 9	57	Male	Right mesial temporal	2.65	15.71	Unmethylated	RTK II

Note. All nine tumors were IDH-wildtype. BOLD MRI was performed on the evening before surgery in every patient. Volumes were derived from anatomical segmentation. Case 5 had no clinically identified enhancing compartment. Molecular findings describe the clinical tumor diagnosis and were not established specifically in the hotspot specimens. Unavailable indicates that no definite methylation class was recorded. BOLD, blood oxygenation level-dependent, CE, contrast-enhancing, FLAIR, fluid-attenuated inversion recovery, IDH, isocitrate dehydrogenase, MRI, magnetic resonance imaging. MGMT, O6-methylguanine-DNA methyltransferase, RTK, receptor tyrosine kinase

### Technical feasibility and target-level neuropathology

Neuronavigation transfer succeeded in all nine cases (100%). Eight candidates were considered accessible during preoperative planning and all eight were sampled (8/9, 88.9%). The ninth was not sampled because no safe trajectory was available. Routine neuropathology documented tumor in seven of eight sampled targets (87.5%). The remaining target yielded cortex and white matter with reactive glial changes and no definite tumor. No target-sampling complications were observed among the eight sampled procedures (0/8) (Table 2).

**Table 2:** Technical feasibility and neuropathology outcomes of high-response habitat-guided biopsy

Case	Navigation transfer successful	Target safely accessible	Target sample obtained	Tumor documented in sampled target on routine neuropathology	Target-sampling complication
Case 1	Yes	Yes	Yes	Yes	None
Case 2	Yes	Yes	Yes	Yes	None
Case 3	Yes	Yes	Yes	Yes	None
Case 4	Yes	Yes	Yes	Yes	None
Case 5	Yes	Yes	Yes	Yes	None
Case 6	Yes	Yes	Yes	Yes	None
Case 7	Yes	Yes	Yes	Yes	None
Case 8	Yes	Yes	Yes	No	None
Case 9	Yes	No	No	NA	NA
**Overall**	**9/9 (100%)**	**8/9 (88.9%)**	**8/9 (88.9%)**	**7/8 (87.5%)**	**0/8 (0%)**

Note. Percentages use all nine cases unless otherwise indicated. Neuropathology and target-sampling complication denominators comprise eight sampled targets and eight procedures, respectively. Tumor-positive status required explicit documentation of tumor in a target specimen. Case 8 was classified as without definite tumor according to the diagnostic report, which also listed a tumor-content estimate < 5% (Supplementary Table S1). Routine neuropathology does not validate tissue hypoxia. BOLD, blood oxygenation level-dependent, NA, not applicable

In the retrospective analysis of the sign-corrected, non-WM-normalized steady-state maps, all eight recorded sampling coordinates fell within the patient-specific Otsu high-response class. Median target-associated habitat volume was 10.25 cm3 (IQR, 4.66–16.87, range, 1.59–21.37). Median target-site DTP was 15.94 s (IQR, 13.84–16.72), median DTR was 13.64 s (IQR, 9.33–16.03), and median lag was 3.31 s (IQR, 0.14–5.32). Among seven patients with enhancement, median target-to-enhancement distance was 3.74 mm (IQR, 2.72–6.33, range, 1.73–9.43) (Table 3).

**Table 3:** Quantitative characterization of the sampled physiological targets

Case	Otsu-defined habitat volume, cm³	Target-to-threshold ratio	Response percentile	Target DTP, seconds	Target DTR, seconds	Target lag, seconds	Distance from target to CE, mm
Case 1	16.73	2.23	99.43	16.73	12.61	3.03	3.00
Case 2	17.28	2.27	99.01	12.98	9.56	0.18	9.43
Case 3	21.37	2.17	97.77	14.12	16.20	3.58	8.54
Case 4	1.87	1.54	96.53	15.58	15.98	6.39	3.74
Case 5	6.49	1.63	93.37	16.30	14.66	4.96	Not applicable
Case 6	1.59	1.49	96.52	16.71	8.60	0.00	4.12
Case 7	5.59	1.43	93.10	23.53	16.26	7.06	2.45
Case 8	14.01	3.02	99.33	9.61	8.62	0.00	1.73

Note. All measurements refer to the recorded sampling coordinate, not the planned target. Target-to-threshold ratios and empirical response percentiles were calculated retrospectively from finite, sign-corrected, non-WM-normalized steady-state values within each patient’s non-enhancing FLAIR mask. Case numbering matches Supplementary Table S1. DTP, DTR, and lag were converted using 1 TR = 1.8 s. Habitat volume represents the 26-connected Otsu high-response component containing the recorded coordinate. Distance to CE was measured in three dimensions to the nearest CE-mask voxel center. Case 5 had no clinically identified enhancing compartment. Distance to CE was therefore not calculated. CE, contrast-enhancing, DTP, delay to plateau, DTR, delay to return, TR, repetition time

The seven tumor-positive targets ranged from infiltrating tumor in adjacent gray and white matter to tumor-rich tissue with reported tumor content or viable tumor-cell estimates up to 80%. Target-specific Ki-67 estimates ranged from approximately 6% to 30%. Necrosis was reported absent in two tumor-positive targets (Cases 2 and 5). Case 8 was a temporobasal cortical target with associated FLAIR hyperintensity. At the recorded sampling location, the response was 3.02 times the retrospectively calculated Otsu threshold and at the 99.33rd percentile within the non-enhancing FLAIR mask. Its specimen showed reactive cortex and white matter without definite tumor, Ki-67 < 1%, and no vascular proliferation. The report also listed tumor content < 5%. For the seven tumor-positive targets, target-to-threshold ratios ranged from 1.43 to 2.27 and response percentiles from 93.10 to 99.43 (Table 3). Supplementary Table S1 presents each target separately and distinguishes unavailable findings from reported absence.

### Representative spatial translation

**Fig. 2:**
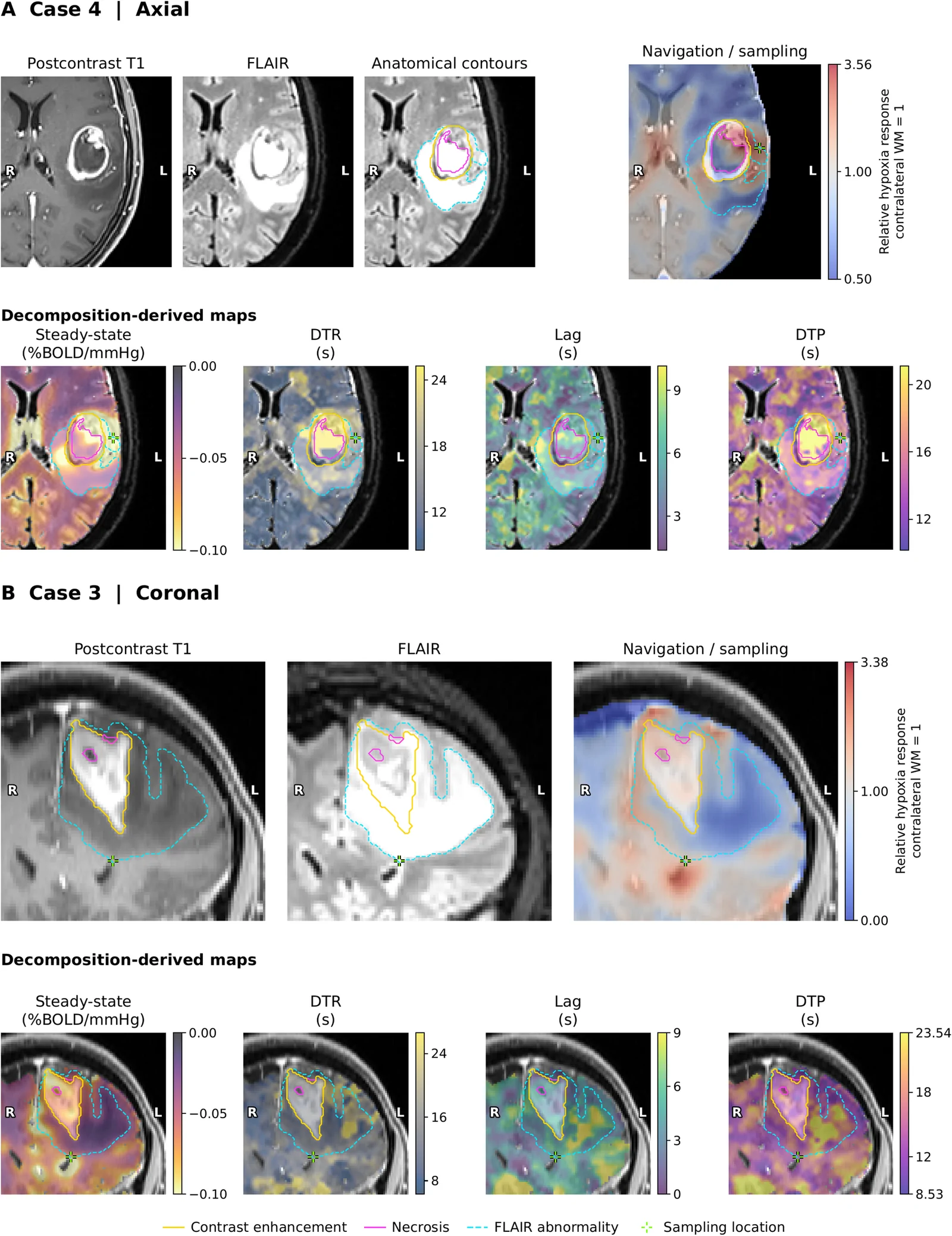
Imaging basis for habitat-guided biopsy in (**A**) Case 4 and (**B**) Case 3. Postcontrast T1-weighted and FLAIR images show the anatomical context of the normalized steady-state response used to guide targeting. Green open-centre crosses indicate the recorded sampling location. Yellow contours indicate the outer border of contrast enhancement. Magenta contours indicate necrosis. Dashed cyan contours indicate the outer FLAIR border. Navigation maps show the response relative to normal-appearing contralateral white matter (WM = 1). Decomposition maps show the non-WM-normalized steady-state response (%BOLD/mmHg), delay to return (DTR), lag, and delay to plateau (DTP). More negative steady-state values indicate a larger BOLD decrease during hypoxia. Timing values are shown in seconds. DTR, lag, and DTP provide complementary temporal context and did not determine target selection. Display limits are indicated by the individual colorbars. Values outside these limits are shown with the endpoint colors. BOLD, blood oxygenation level-dependent, FLAIR, fluid-attenuated inversion recovery

Figure 2 shows two representative cases. Green markers indicate the recorded sampling locations outside contrast enhancement. The normalized response map shows the high-response region used for targeting. The non-WM-normalized steady-state map and the DTR, lag, and DTP maps provide complementary context. Timing maps did not define the target.

## Discussion

We prospectively translated a patient-specific high-response hypoxia-powered MRI habitat beyond contrast enhancement into a coordinate-matched surgical tissue target. Map generation and neuronavigation transfer succeeded in all nine patients. We sampled eight targets. Seven contained tumor, and we observed no target-sampling complications. We did not sample the ninth target because no safe trajectory was available. Surgical teams can integrate a tracer-free, stimulus-evoked physiological map into planning and use it to sample prospectively defined targets in the non-enhancing compartment.

The value of the workflow lies in preserving a link among the physiological signal, the recorded sampling location, and the tissue result. The workflow follows recommendations for biological imaging, neuronavigation, early sampling, spatial documentation, and coordinated tissue processing [11]. We defined the habitat quantitatively and patient-specifically before pathology results were available, transferred it to neuronavigation, and sampled it before major debulking in every sampled case. Each specimen retained a target-specific pathology identifier, and we divided each specimen into formalin-fixed, paraffin-embedded and frozen tissue, supporting morphology, immunohistochemistry, and future molecular analyses [11, 21]. Because freezing delay and cross-disciplinary coordination affect downstream readouts, this linkage also provides a framework for controlled spatial biobanking [22, 23].

Seven of eight prospectively selected high-response habitats contained tumor beyond the enhancing contour. This is a preliminary observation in selected, surgically accessible targets. Without matched low-response FLAIR samples, it does not establish tumor enrichment, diagnostic accuracy, or improved detection over FLAIR-based targeting. Our tumor-positive samples add to previous spatial sampling evidence that non-enhancing regions contain viable tumor and regionally distinct biology [7, 8, 24]. Cepeda et al. recently provided prospective paired-biopsy and transcriptomic validation of an artificial intelligence-derived infiltration-risk map [12]. Their findings establish the value of spatial infiltration mapping beyond enhancement. The present study addresses a complementary question by using a controlled stimulus to derive a tracer-free physiological target and carrying that target into surgery. Its contribution is therefore not the first imaging-guided biopsy beyond enhancement, but, to our knowledge, the first prospective surgical translation and tissue anchoring of hypoxia-powered MRI habitats.

The sample without definite tumor was also informative. This temporobasal cortical target showed FLAIR hyperintensity and contained reactive cortex and white matter without definite tumor, illustrating that non-enhancing FLAIR abnormality is not synonymous with tumor infiltration. This compartment can include edema, infiltrative tumor, reactive tissue, and admixed cortex. A purely volumetric FLAIR target therefore cannot distinguish tissue that may warrant treatment from tissue that may not. The response at the recorded sampling location was well above the retrospectively calculated Otsu threshold, rather than borderline. The case therefore also shows that a prominent BOLD response does not establish tumor specificity. Cortical vascular contributions and partial-volume effects may have contributed to the response relative to white matter [16]. The temporobasal location also raises the possibility of EPI distortion or signal loss near air-tissue interfaces [25]. Registration error or sampling mismatch remain possible. Routine neuropathology did not determine the physiological basis of the response. Crucially, the retained image-to-tissue linkage exposes this discordance and shows how a larger paired-sampling study can test it.

The tumor-positive targets also showed heterogeneity. Ki-67 ranged from approximately 6% to 30%, and necrosis was reported absent in two tumor-positive targets. We did not assess correlations with response magnitude because eight selected targets and non-standardized routine pathology estimates do not support a reliable between-patient analysis. This question requires a larger cohort with standardized, spatially matched pathology. Metabolic positron emission tomography sampling has identified higher neoplastic cell density and mitotic activity in metabolic hotspots than in FLAIR-only tissue [26], while spatial immunosampling has shown distinct immune composition at non-enhancing margins [27]. Rather than reducing the non-enhancing compartment to a binary tumor versus non-tumor label, the linked specimens we collected here provide a substrate for testing whether hypoxia-powered MRI habitats correspond to distinct vascular, hypoxia-related, immune, or molecular states.

Taken together, the tumor-positive and tumor-negative results illustrate why functional partitioning of the non-enhancing compartment matters. Current definitions of FLAIR-based supramaximal resection focus mainly on the volume of abnormality removed, while outcome evidence remains retrospective and heterogeneous [3, 28]. A more clinically relevant question is not only how much FLAIR abnormality to treat, but which regions contain tumor or treatment-relevant biology. Patient-specific physiological habitats could ultimately support selective biopsy or resection, radiation dose painting, and local drug delivery. The present study provides the required navigation and tissue-anchoring framework, but not yet evidence for treatment selection.

This proof-of-concept study has the following limitations. The nine-patient cohort and eight sampled targets cannot estimate yield or safety with precision. Sampling one accessible hotspot region per patient favors surgically reachable regions and does not capture within-patient heterogeneity. Otsu thresholding defined the candidate, while surgical accessibility and the final sampling point required clinical judgment. Interobserver reproducibility was not assessed. We did not obtain matched low-response FLAIR controls, transcriptomic profiling, or a diagnostic-accuracy analysis. Available main-tumor specimens were routine clinical comparators. Neuropathologists were not blinded to BOLD findings, introducing potential assessment bias. Routine neuropathology confirmed tumor presence but did not validate tissue hypoxia or define the vascular and molecular basis of the response. Registration underwent visual quality control, but localization accuracy was not formally quantified. Echo-planar distortion, partial-volume effects, image registration, neuronavigation error, and brain shift add spatial uncertainty despite sampling before major debulking. Observational complication capture also limits safety inference. The results therefore establish operational feasibility and target-specific tissue linkage, not diagnostic specificity or procedural safety.

The next validation step follows directly from the observed heterogeneity. Future studies should prespecify the habitat definition and obtain spatially matched samples from the high-response habitat, low-response FLAIR tissue, and the enhancing compartment within each patient. Multiple samples per compartment and blinded, standardized pathology would permit assessment of biological enrichment. Estimating sensitivity and specificity would additionally require an appropriate sampling design and reference standard.

Coordinated analysis of formalin-fixed, paraffin-embedded and frozen material should assess cellularity, proliferation, vascular architecture, hypoxia-related pathways, immune composition, and molecular state. Spatial transcriptomics, proteomics, and metabolomics could determine whether the habitats correspond to reproducible biological states. Multimodal MRI may help separate oxygenation-related responses from perfusion and cortical effects. Longitudinal follow-up can then test whether these habitats relate to recurrence [29] or response to spatially targeted treatment [30].

In conclusion, the GLIOXY initiative establishes an imaging-to-tissue pathway for prospective sampling of hypoxia-powered MRI habitats beyond contrast enhancement. Seven of eight sampled habitats contained tumor. By linking a tracer-free, stimulus-evoked physiological map to recorded sampling locations and tissue, this study moves hypoxia-powered MRI from descriptive imaging toward prospective biological investigation of the non-enhancing glioblastoma compartment.

## Supplementary Information

Below is the link to the electronic supplementary material.

Supplementary Material 1

## Data Availability

The data supporting this study are available from the corresponding author on reasonable request. They are not publicly available because they form part of an ongoing study and include patient-level clinical, imaging, and neuropathological information. Access is subject to the applicable ethics and data-protection framework.
