# Supplementary material for: Hypoxia-powered MRI guides supramaximal biosurgical sampling of glioblastoma infiltration: the GLIOXY initiative
Source: J Neurooncol. 2026 Nov 6;180(2):39. doi: 10.1007/s11060-026-05864-1 (PMC13743575; doi:10.1007/s11060-026-05864-1)
Supplement: Supplementary file 1 [file 11060_2026_5864_MOESM1_ESM.docx]

Supplementary Table S1. Hotspot-specific findings from routine clinical neuropathology

| **Case** | **Hotspot-specific pathology** | **Ki-67** | **Necrosis** | **Vascular proliferation** | **Available clinical comparator or additional finding** |
| --- | --- | --- | --- | --- | --- |
| Case 1 | Tumor-positive. Approximately 60% tumor core with additional infiltration into gray and white matter. | 15–30% | Present | Glomeruloid proliferation present | Enhancing tumor and hotspot had similar Ki-67 and p53 overexpression. Hotspot included an infiltration zone. |
| Case 2 | Tumor-positive. Increased cellularity and numerous mitoses. | ≈30% | Absent in hotspot | Absent in hotspot | Enhancing tumor contained very-cell-dense areas with vascular proliferation and necrosis. Hotspot was highly proliferative but viable/non-necrotic. |
| Case 3 | Tumor confirmed in the periventricular deep hotspot specimen. | 12% | Not reported for hotspot | Not reported for hotspot | Main tumor Ki-67 was 8%, focally 20%. Approximately 25% necrosis was attributed to the main tumor. |
| Case 4 | Tumor-positive. CNS-infiltrating tumor in tissue containing adjacent gray and white matter. | ≈10% | Not reported | Not reported | Dense tumor core had Ki-67 10-15%. Both a 5-ALA-negative and a 5-ALA-positive specimen from the same hotspot region contained infiltrating tumor. |
| Case 5 | Approximately 80% viable tumor cells. | Up to 10% | Absent | Focal proliferation present | Main tumor: viable tumor 35% / Ki-67 5%. Hippocampal tumor: 36% / 5%. |
| Case 6 | Dense tumor core. Morphologically similar to enhancing tumor. | ≈6% | 5% | Not reported | Enhancing tumor had 20% necrosis and Ki-67 6%. Hotspot was less necrotic. |
| Case 7 | Tumor-positive with 80% reported tumor content and high proliferative activity. p53 overexpression in 80%. | Up to 20% | 40% | Not reported | Enhancing tumor and hotspot received essentially identical quantitative summaries. |
| Case 8 | No definite tumor. Reactive cortex and white matter. The report also listed tumor content <5%. | <1% | Absent / not reported as tumor necrosis | Absent | Enhancing tumor contained 80% tumor, vascular proliferation, necrosis, and Ki-67 20%. |

Note. Findings were abstracted only when explicitly attributed to the targeted specimen. Original qualifiers such as approximately, up to, and ranges were retained. Not reported does not indicate absence. Routine pathology was not blinded to blood oxygenation level-dependent (BOLD) findings. Main-tumor comparator findings were available clinical observations, not systematic spatially matched control biopsies. Percentages of tumor content and necrosis were retained as reported. Their denominators were not consistently specified and should not be assumed to be identical. CNS, central nervous system, 5-ALA, 5-aminolevulinic acid.
